# Identification and Validation of a Prognostic Signature for Prostate Cancer Based on Ferroptosis-Related Genes

**DOI:** 10.3389/fonc.2021.623313

**Published:** 2021-07-15

**Authors:** Huan Liu, Lei Gao, Tiancheng Xie, Jie Li, Ting-shuai Zhai, Yunfei Xu

**Affiliations:** ^1^ Department of Urology, Shanghai Tenth People’s Hospital, School of Medicine in Tongji University, Shanghai, China; ^2^ Department of Urology, The Second Hospital of Hebei Medical University, Shijiazhuang, China; ^3^ Department of Orthopedics, Jingan District Zhabei Central Hospital, Shanghai, China

**Keywords:** ferroptosis, recurrence free survival, TFRC, gene signature, prostate cancer

## Abstract

Ferroptosis, an iron-dependent form of selective cell death, is involved in the development of many cancers. However, ferroptosis related genes (FRGs) in prostate cancer (PCa) are not been well studied. In this study, we collected the mRNA expression profiles and clinical information of PCa patients from TCGA and MSKCC databases. The univariate, LASSO, and multivariate Cox regression analyses were performed to construct a prognostic signature. Seven FRGs, *AKR1C3*, *ALOXE3*, *ATP5MC3*, *CARS1*, *MT1G*, *PTGS2*, and *TFRC*, were included to establish a risk model, which was validated in the MSKCC dataset. The results showed that the high-risk group was apparently correlated with copy number alteration load, tumor burden mutation, immune cell infiltration, mRNAsi, immunotherapy, and bicalutamide response. Moreover, we found that *TFRC* overexpression induced the proliferation and invasion of PCa cell lines *in vitro*. These results demonstrate that this risk model can accurately predict prognosis, suggesting that FRGs are promising prognostic biomarkers and potential drug targets in PCa patients.

## Introduction

Prostate cancer (PCa) is one of the world’s most common malignancies in men, and is the second-leading cause of cancer-related deaths in Western countries (1). Prostate-specific antigen (PSA) has been used as the standard PCa detection test since the 1990s. However, previous studies found no significant differences in mortality between PSA-screened patients and those who are not screened (2–4). PSA is also known to be a major predictor of PCa prognosis. Nearly 27-53% of patients who have undergone radical prostatectomy and radiotherapy experience PSA recurrence (5). Such biochemical recurrence (BCR) can contribute to the development of advanced castration-resistant prostate cancer (CRPC) stage, leading to an increased risk in distant metastases, prostate cancer-specific mortality and overall mortality (6, 7). Therefore, it is of great significance to identify novel prognostic biomarkers for PCa.

Ferroptosis, a newly identified form of regulated cell death characterized by iron accumulation and lipid peroxidation, is distinct from other forms of regulated cell death (necroptosis, apoptosis, or autophagic cell death) (8). Recently, emerging evidence suggested that ferroptosis is related to cancer initiation, progression, or drug sensitivity (9–11). Ferroptosis inducers can be used to resolve drug resistance and prevent cancer progression or metastasis. For example, Sun et al. reported that metallothionein-1G (*MT-1G*) knockdown enhanced sorafenib sensitivity in hepatocellular carcinoma *via* promoting ferroptosis (10). It has been shown that suppression of cysteine dioxygenase 1 (*CDO1*) increases cellular glutathione (*GSH*) levels, inhibits reactive oxygen species (*ROS*) generation, and decreases lipid peroxidation in erastin-treated gastric cancer cells (12).

The role of ferroptosis in PCa has drawn attention in recent years. Butler et al’s study revealed that DECR1 knockdown in PCa cells inhibited tumor cell proliferation and migration *via* cellular polyunsaturated fatty acids (PUFAs) accumulation, thus enhancing mitochondrial oxidative stress and lipid peroxidation, and promoting ferroptosis (13). Pannexin 2 (*PANX2*) has been proposed to be a new marker in PCa, that promotes proliferation and invasion of PCa cells through regulating ferroptosis (14). While preliminary evidence has identified several markers that are correlated with PCa ferroptosis, the association between other ferroptosis-related genes (FRGs) and PCa prognosis remains largely unknown.

In our research, we analyzed the mRNA expression profiles of 40 ferroptosis-related genes and clinical data of PCa patients from the TCGA database. We then evaluated their differential expression in different risk PCa samples and investigated the enriched pathways and biological roles. Next, a FRGs-based prognostic model were constructed in the TCGA dataset and validated it in another dataset. Moreover, we chose the hub gene transferrin receptor (*TFRC*) for further validation *in vitro*.

## Materials and Methods

### Data Collection

The RNA-seq (FPKM value) data of 499 PCa and 52 normal prostate tissues with related clinical data were obtained from the TCGA website (https://portal.gdc.cancer.gov/repository). The MSKCC data set downloaded from the GEO dataset (https://www.ncbi.nlm.nih/geo/query/), containing genomic profile of 218 prostate tumors was used as the validation cohort

### Gene Signature Building

A total of 40 genes from Stockwell et al’s research were analyzed in the current study (15) (shown in **Supplementary Table S1**). Univariate Cox was performed to select genes related to the recurrence-free survival (RFS), using P values less than 0.05 as the cut-off. The LASSO method was used to minimize the risk of overfitting. Finally, multiple stepwise Cox regression was used to establish the risk model. The risk score of hub genes was established as (exprgene1 × coefficientgene1) + (exprgene2 × coefficientgene2) + ⋯ + (exprgene7 × coefficientgene7).

### Clustering, Genetic Alterations, and Functional Enrichment Analysis

Consensus clustering based on ferroptosis genes was achieved using the “Consensus ClusterPlus” package (16). The “clusterProfiler” R package (17) was used to perform GO and KEGG analyses based on differentially expressed genes (DEGs) (|log2FC| ≥ 1, FDR < 0.05) between different risk groups. The infiltrating score of 28 immune cells was calculated with ssGSEA in the “GSVA” R package (18). The annotated gene set file is shown in **Supplementary Table S2**. Genetic alterations of selective genes in TCGA patients were acquired from the cBioPortal (19, 20). The relationships between mRNA expression level and Gleason score, lymph nodal metastasis, methylation levels were obtained from UALCAN (21).

### Copy Number Variation (CNV) Load, Tumor Mutation Burden (TMB), Neoantigens, Tumor Stemness, and Clonal Score

The TCGA data was divided into two risk groups based on the median risk value. The CNV load at the focal and arm levels were then calculated based on the GISTIC_2.0 results, which were freely available from Broad Firehose (https://gdac.broadinstitute.org/). Each patient’s tumor mutation burden (TMB) was measured as the total number of non-synonymous mutations per megabase. Tumor neoantigens, which can be recognized by neoantigen-specific T cell receptors (TCRs) and participate in T-cell-mediated antitumor immune response, were analyzed using the TCIA (https://tcia.at/). We also analyzed the clonality of PCa patients from the TCIA dataset (Clonality is the critical character of tumors). Considering that tumor stemness is also one of the basic characteristics of the tumor, the mRNAsi data was analyzed as reported by Robertson (22).

### Prediction of Immunotherapy and Chemotherapy Responses

The Tumor Immune Dysfunction and Exclusion (TIDE) and subclass mapping (23–25) were performed to assess the clinical response of immune therapy in PCa patients. Chemotherapeutic response to three commonly used drugs for PCa patients in the TCGA and MSKCC datasets was calculated using the Genomics of Drug Sensitivity in Cancer (GDSC, https://www.cancerrxgene.org) *via* performing ‘pRRophetic’ R package (26).

### Cell Culture, TFRC Stably Over-Expressed Cell Lines Establishment and Reagents

The HEK293T cell line, and prostate cancer cells C4-2, PC-3, LNCaP, 22RV1 were purchased from American Type Culture Collection (ATCC, Manassas, VA). HEK293T cells were cultured in a humidified atmosphere (37°C with 5% CO_2_) in DMEM whereas prostate cancer cells were grown in RPMI1640. 22RV1 and LNCaP cells were treated with 5-Azacytidine (Sigma, #A2385). The TFRC overexpressing plasmid was designed and synthesized by Genomeditech (Shanghai, China). The TFRC overexpression plasmids, psPAX2 and pMD2.G were mixed and added into HEK293T cells to package lentivirus. After incubation for 48 h, lentivirus supernatants were collected, filtered, and used to infect cells.

### Western Blot Analysis

The cells were washed thrice with cold PBS buffer and lysed with RIPA buffer on ice. We loaded the same amounts (40 µg) of different protein samples onto the SDS-PAGE gel and then transferred the protein to PVDF membranes. The membranes were blocked using skim milk (5%) for 1 h and incubated with primary antibodies against TFRC (ABclonal, #A5865) or β-Actin (sc-517582) at 4°C overnight. The next day, we incubated the PVDF membranes with HRP-conjugated secondary antibodies (mouse or rabbit) at room temperature for 1h. The blots were visualized using an ECL system (Thermo Fisher Scientific) after washing thrice using TBST.

### Cell Invasion Assay

We used transwell chambers (8 μm pore size, Corning, MA, USA) to detect the migration and invasion of 22RV1 and LNCaP after TFRC overexpression. The upper chamber was pre-coated with Matrigel (Corning, USA) for invasion and was left uncoated for migration. Next, 10^5^ cells were seeded in serum-free media in the upper chamber, while 600 μL of culture media containing was added in the lower chamber. After 12 h (migration) or 24 h (invasion), we used a cotton swab to remove the cells remaining in the upper surfaces of the chambers and fixed the cells on the lower surface in 100% methanol for 15 min. We then stained them using 0.1% crystal violet solution for 20 min. The cells were counted and averaged across five randomly chosen fields using a microscope.

### Statistics

All data analyses were performed using the R platform (v.4.0.2, https://cran.r-project.org/) or GraphPad Prism 8.0. The comparison of mRNA expression between PCa tissues and adjacent nontumorous samples, CNV, TMB, mRNAsi, clonal score, chemotherapy response and immunotherapy response across different risk groups were calculated using the Wilcoxon rank-sum test. The RFS of different groups was measured using Kaplan-Meier log-rank test method. The DEGs between different risk groups (high and low) were determined utilizing the “limma” R package (27). The results of MTT, invasion and migration results were analyzed using Student’s t test. *P* < 0.05 was considered as statistically significant.

## Results

### Expression and Correlation of FRGs in the TCGA Cohort

**Figure 1:**
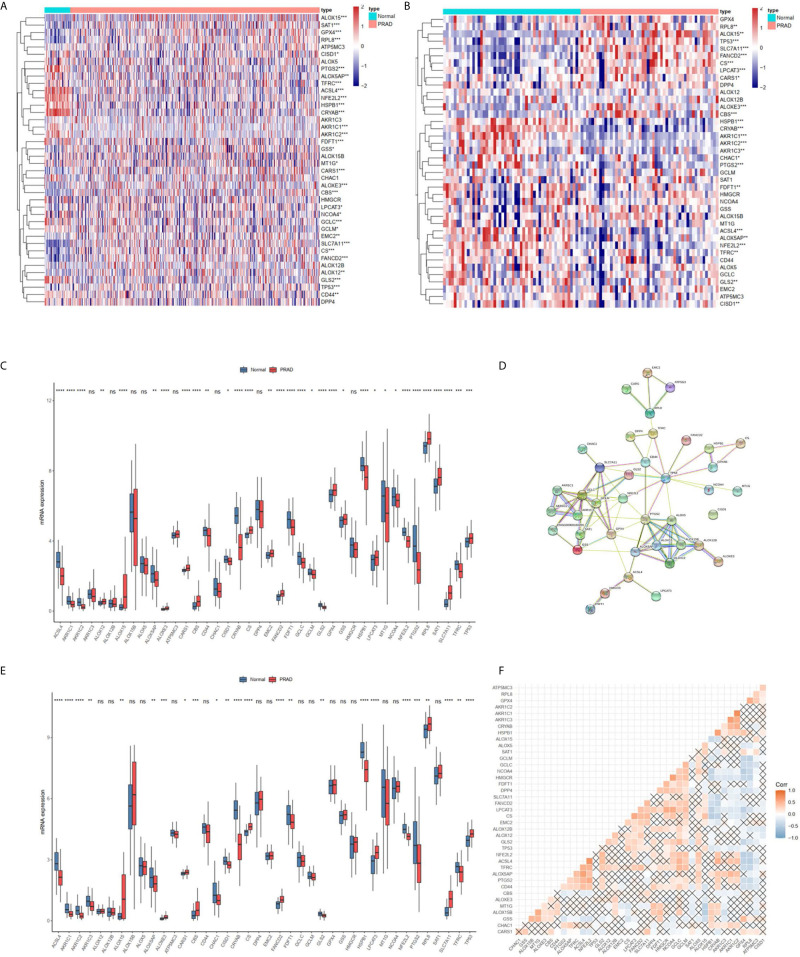
The landscape of ferroptosis-related genes (FRG) in prostate cancer. **(A)** Heatmap of 40 FRGs between 499 prostate cancer and normal tissues. **(B)** Heatmap of 40 FRGs between 52 tumor and adjacent normal pairs. **(C)** mRNA expression levels FRGs levels in total prostate cancer and normal tissues. **(D)** The PPI network acquired from the STRING database among the FRGs. **(E)** mRNA expression levels FRGs levels in prostate cancer and paired tissues. **(F)** The correlation among FRG in prostate cancer. *P < 0.05, **P < 0.01, ***P < 0.001, ****P < 0.0001. ns, not significant.

Most FRGs (32/40, 80%) (**Figures 1A, C** **)**, (24/40,60%) (**Figures 1B, E** **)** in the TCGA dataset were abnormally expressed in tumor samples compared with adjacent nontumorous tissues, and paired nontumorous tissues. The interaction network among these 40 genes demonstrated that *TP53*, *GCLC*, *GCLM*, *GPX4* and other genes had more interactions (**Figure 1D**). Moreover, the correlations between these genes were very high (**Figure 1F** **)**, for example, *ACSL4* and *NFE2L2*, *HMGCR* and *NCOA4*, *HSPB1* and *CRYAB*.

### Clustering, Construction and Validation of FRGs Prognostic Model

**Figure 2:**
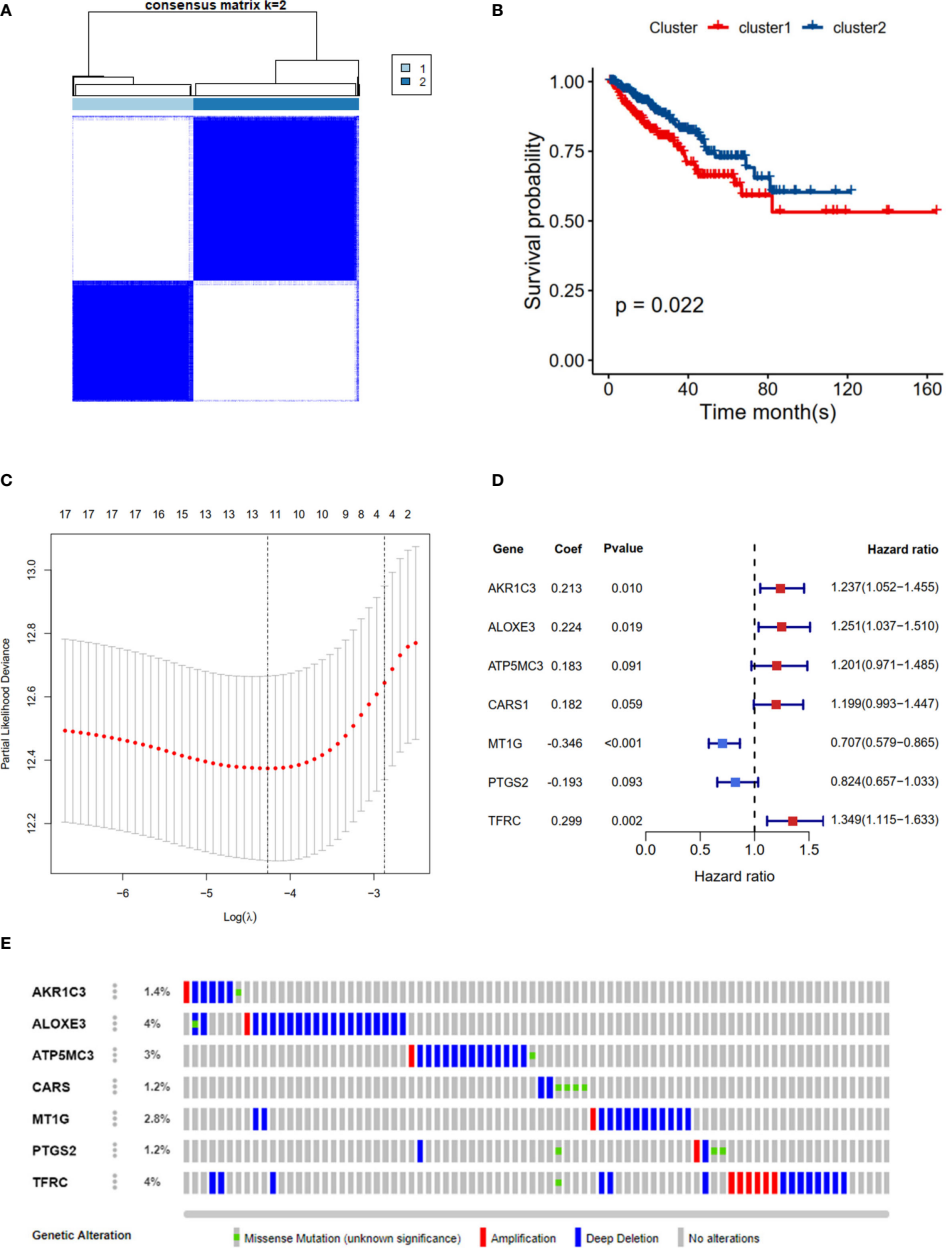
RFS of PRAD patients in cluster 1/2 subgroups and risk signature with 7 FRGs. **(A)** Consensus Clustering matrix for k =2. **(B)** Kaplan-Meier curves of DFS. **(C)** The Cross-Validation fit curve calculated by lasso regression method. **(D)** The coefficients of seven hub FRGs estimated by multivariate Cox regression. **(E)** Genetic alterations of seven hub FRGs. All the survival analysis are based on the recurrence free survival data.

The TCGA cohort could be divided into two clusters according to the mRNA expression levels of the 40 FRGs (**Figure 2A**). Patients in cluster 1 had significantly poorer RFS than those in cluster 2 (**Figure 2B**), suggesting that FRGs were associated with differences between PCa patients. Therefore, identifying prognostic FRGs was necessary. The univariate Cox regression method was first used to identify FRGs that were associated with prognosis; 17 genes were found to be correlated with PCa RFS (**Table 1**). The LASSO method was then used for selecting the hub genes (**Figure 2C**) to minimize the risk of overfitting. To further identify the FRGs with the greatest prognostic value, we conducted multiple stepwise Cox regression and chose seven hub FRGs to construct the prognostic model of PCa patients (**Figure 2D**). These seven hub genes had greater than 1% genetic alterations, for example *TFRC* (4%) and *ALOXE3* (4%) (**Figure 2E**). The risk scores of each PCa patient were measured using the following method: Risk score = (0.213*ExpAKR1C3) + (0.224*ExpALOXE3) + (0.183*ExpATP5MC3) + (0.182*ExpCARS1) + (-0.346*ExpMT1G) + (-0.193*ExpPTGS2) + (0.299*ExpTFRC). We then divided the PCa patients of the TCGA cohorts into two groups (high and low risk) according to the median value of the risk score.

**Table 1:** Univariate Cox regression results of 40 ferroptosis related genes.

id	HR	95%CI	pvalue	id	HR	95%CI	pvalue
ACSL4	0.95	0.78-1.16	0.625	GCLC	1.09	0.89-1.35	0.397
AKR1C1	1.28	1.10-1.49	0.001	GCLM	1.16	0.95-1.41	0.153
AKR1C2	1.11	0.91-1.36	0.293	GLS2	1.03	0.84-1.28	0.756
AKR1C3	1.42	1.21-1.66	0.001	GPX4	1.22	1.00-1.50	0.054
ALOX5	1.09	0.89-1.35	0.389	GSS	0.97	0.79-1.19	0.777
ALOX12	1.23	1.04-1.45	0.015	HMGCR	0.85	0.70-1.03	0.097
ALOX15	1.05	0.86-1.28	0.657	HSPB1	0.95	0.78-1.17	0.651
ALOXE3	1.25	1.05-1.50	0.013	CRYAB	0.78	0.63-0.97	0.027
ALOX12B	1.19	0.98-1.44	0.075	LPCAT3	0.88	0.73-1.06	0.170
ALOX15B	0.75	0.61-0.91	0.004	MT1G	0.65	0.53-0.79	0.001
ALOX5AP	1.21	0.99-1.48	0.056	NCOA4	0.84	0.69-1.02	0.081
ATP5MC3	1.3	1.04-1.63	0.02	NFE2L2	0.88	0.73-1.05	0.159
CARS1	1.32	1.09-1.59	0.004	PTGS2	0.78	0.62-0.97	0.026
CBS	1.09	0.89-1.34	0.401	RPL8	1.09	0.89-1.33	0.396
CD44	0.82	0.68-0.99	0.034	SAT1	1.21	0.99-1.48	0.061
CHAC1	1.17	0.98-1.40	0.089	SLC7A11	1.09	0.89-1.34	0.400
CISD1	0.93	0.76-1.15	0.515	FDFT1	0.89	0.73-1.1	0.280
CS	1.27	1.03-1.57	0.025	TFRC	1.36	1.12-1.66	0.002
DPP4	0.68	0.57-0.82	0.001	TP53	0.81	0.69-0.95	0.009
FANCD2	1.43	1.20-1.70	0.001	EMC2	1.25	1.04-1.51	0.017

**Figure 3:**
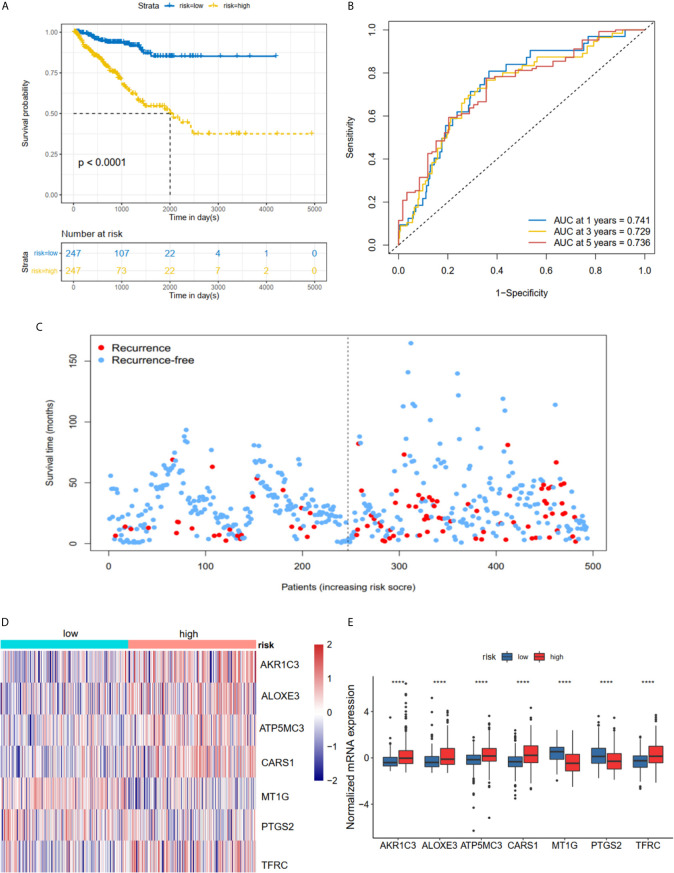
Risk score based on seven hub FRGs in TCGA PRAD cohort. **(A)** Survival analysis according to risk score. **(B)** ROC analysis. **(C)** Survival status of patients. **(D, E)** Heatmap and barplot of the seven hub genes between high and low risk group. All the survival analysis are based on the recurrence free survival data. ****P < 0.0001.

**Figure 4:**
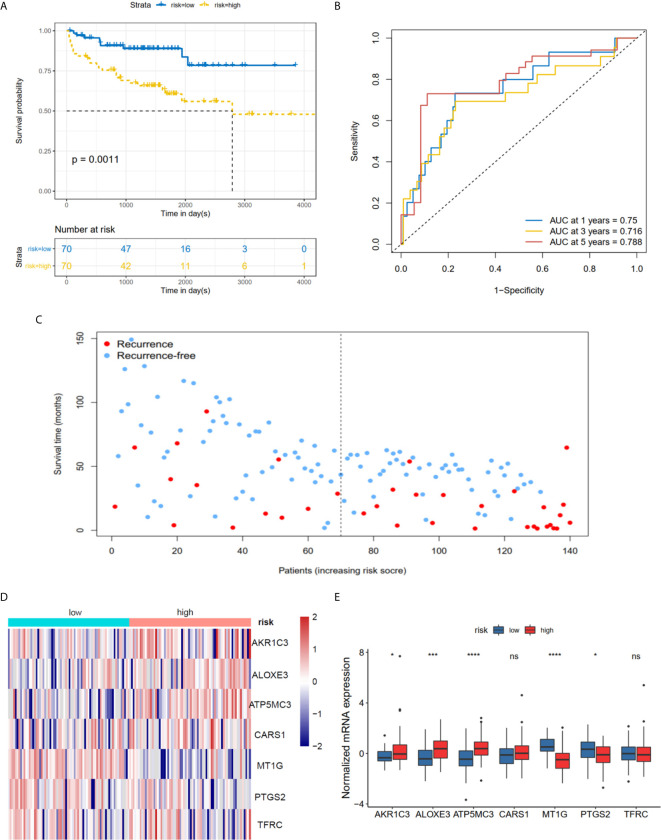
Risk score based on seven hub FRGs in MSKCC PRAD cohort. **(A)** Survival analysis according to risk score. **(B)** ROC analysis. **(C)** Survival status of patients. **(D, E)** Heatmap and barplot of the seven hub genes between high and low risk group. All the survival analysis are based on the recurrence free survival data. *P < 0.05, ***P < 0.001, ****P < 0.0001. ns, not significant.

The K-M plot demonstrated that the high-risk group had unfavorable RFS compared with the low-risk group (P < 0.0001, **Figure 3A**). Moreover, the area under the receiver operating characteristic curve (AUC) for 1-year, 3-year and 5-year RFS were 0.741, 0.729 and 0.736 (**Figure 3B**), respectively, which were good classification results. The RFS status of PRAD showed that more patients relapsed in the high-risk group (**Figure 3C**). The heatmap and bar plot showed that *AKR1C3*, *ALOXE3*, *ATP5MC3*, *CARS1*, *TFRC* in the high-risk group were increased, while *MT1G* and *PTGS2* were decreased (**Figures 3D, E** **)**. Furthermore, univariate and multivariate Cox regression analysis showed that the risk score was an independent factor RFS for PCa patients in the TCGA cohort (HR = 1.11, 95% CI:1.08-1.15, p value < 0.001, **Table 2**). In the MSKCC dataset, the KM plot results showed that the high risk group had unfavorable RFS, consistent with our observation in the TCGA dataset (HR = 1.76, 95% CI: 1.43-2.17, p < 0.001, **Figure 4A** and **Table 2**). While the associated survival statuses and mRNA expression of these seven genes were somewhat different between risk groups (**Figures 4B–E**), the AUC for the 1-year, 3-year, and 5-year RFS, was greater than 0.7, suggesting that the risk model can be useful in predicting prognosis. Moreover, the risk score of the AUC in the two datasets was marginally higher than that of T status (TCGA:0.731>0.648; MSKCC:0.736>0.468; **Figure S1F**), but this not the case for the Gleason score.

**Table 2:** Univariate Cox regression and multivariate Cox regression analysis of riskScore and clinical information in TCGA and MSKCC cohorts.

Variables	Unicox			Multicox		
	HR	95%CI of HR	P	HR	95%CI of HR	P
**TCGA**						
age	1.00	0.99-1.06	0.192			
Gleason(10/9/8/7/6)	2.20	1.76-2.83	<0.001	2.00	1.52-2.57	<0.001
T(T4/T3/T2)	2.60	1.66-3.95	<0.001	1.08	1.04-1.13	<0.001
N(N1/N0)	1.70	1.03-2.85	0.037	0.80	0.46-1.39	0.425
PSA	1.10	1.03-1.10	<0.001	1.00	0.99-1.07	0.101
riskScore	1.11	1.07-1.15	<0.001	1.89	1.01-3.52	0.044
**MSKCC**						
age	1.02	0.97-1.08	0.381			
Gleason(10/9/8/7/6)	3.7	2.57-5.33	<0.001	3.28	2.22-4.86	<0.001
T(T3/T2/T1)	1.53	0.74-3.15	0.257			
riskScore	1.76	1.43-2.17	<0.001	1.39	1.07-1.79	0.016

### Functional Analyses in TCGA and MSKCC Cohorts

**Figure 5:**
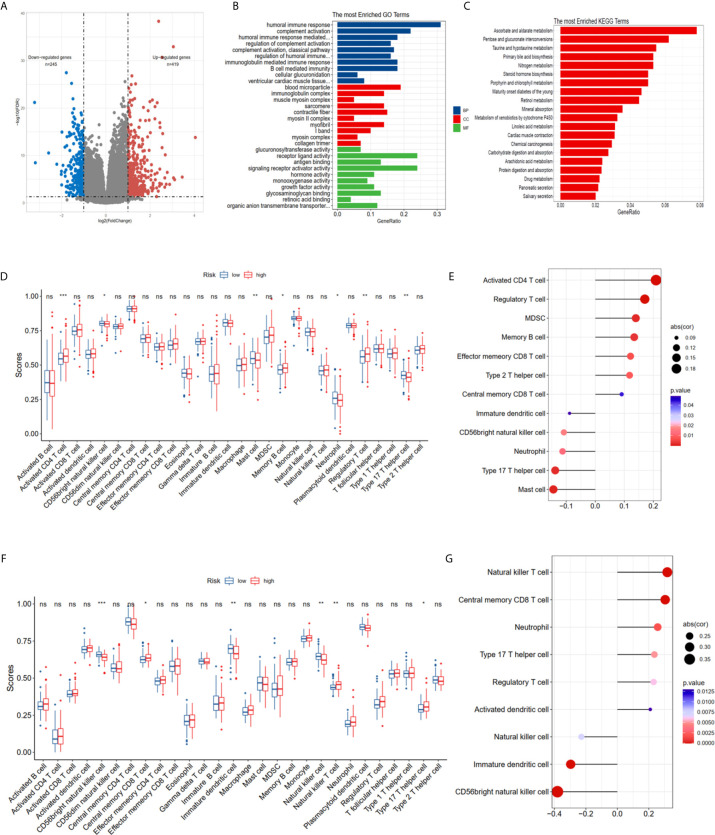
Potential biological pathways affected by FRGs. **(A)** The different expression genes (DEGs) between the high-risk and low-risk groups. **(B)** The Gene Ontology (GO) enrichment of DEGs. **(C)** The KEGG enrichment of high and low risk groups. Comparison and correlations of the ssGSEA scores between different risk groups and risk scores in TCGA **(D, E)** and MSKCC **(F, G)** dataset. *P < 0.05, **P < 0.01, ***P < 0.001. ns, not significant.

The DEGs between the high risk and low risk groups were analyzed in the TCGA-PCa cohort to further clarify the biological functions and pathways of FRGs. The set comprised 664 genes (adjusted P < 0.05, |log2FC| > 1), including 419 upregulated and 245 downregulated genes (**Figure 5A**). GO and KEGG enrichment was then performed using the ClusterProfiler R package. Interestingly, most of the GO terms were enriched for immune-related functions (**Figure 5B**), such as humoral immune response, regulation of humoral immune response, B cell mediated immunity, immunoglobulin complex, antigen binding and growth factor activity. Meanwhile, the KEGG terms were closely associated with several metabolism processes (**Figure 5C**), such as ascorbate and aldarate metabolism, steroid hormone biosynthesis, and especially drug metabolism and xenobiotics metabolism by cytochrome P450, which are essential pathways for the metabolism of many drugs. The statuses of the 28 immune infiltrating cells were then calculated using the ssGSEA method since the risk score was strongly associated with the immune response. The activated CD4 T cell, CD 56^dim^ natural killer cell, mast cell, memory B cell, neutrophil, regulatory T cell and type 17 T helper cell were significantly different in both the low- and high-risk groups in the TCGA dataset (P <0.05, **Figure 5D**). The CD 56^dim^ natural killer cell, central memory CD8 T cell, natural killer cell, natural killer T cell, and type 17 T helper cell were significantly different between groups with low or high risk in the MSKCC dataset (P <0.05, **Figure 5D**). The risk scores were associated with regulatory T cell, type 17 T helper cell, CD 56 bright natural killer cells and neutrophils (**Figures 5E, G** **)**, indicating that FRGs can regulate the progress of PCa *via* the immune process pathway.

### The Distinctions of Gene TMB, CNV, Cancer Stemness Index, and Sensitivity to Immunotherapy/Chemotherapy in the High- and Low-Risk Groups

**Figure 6:**
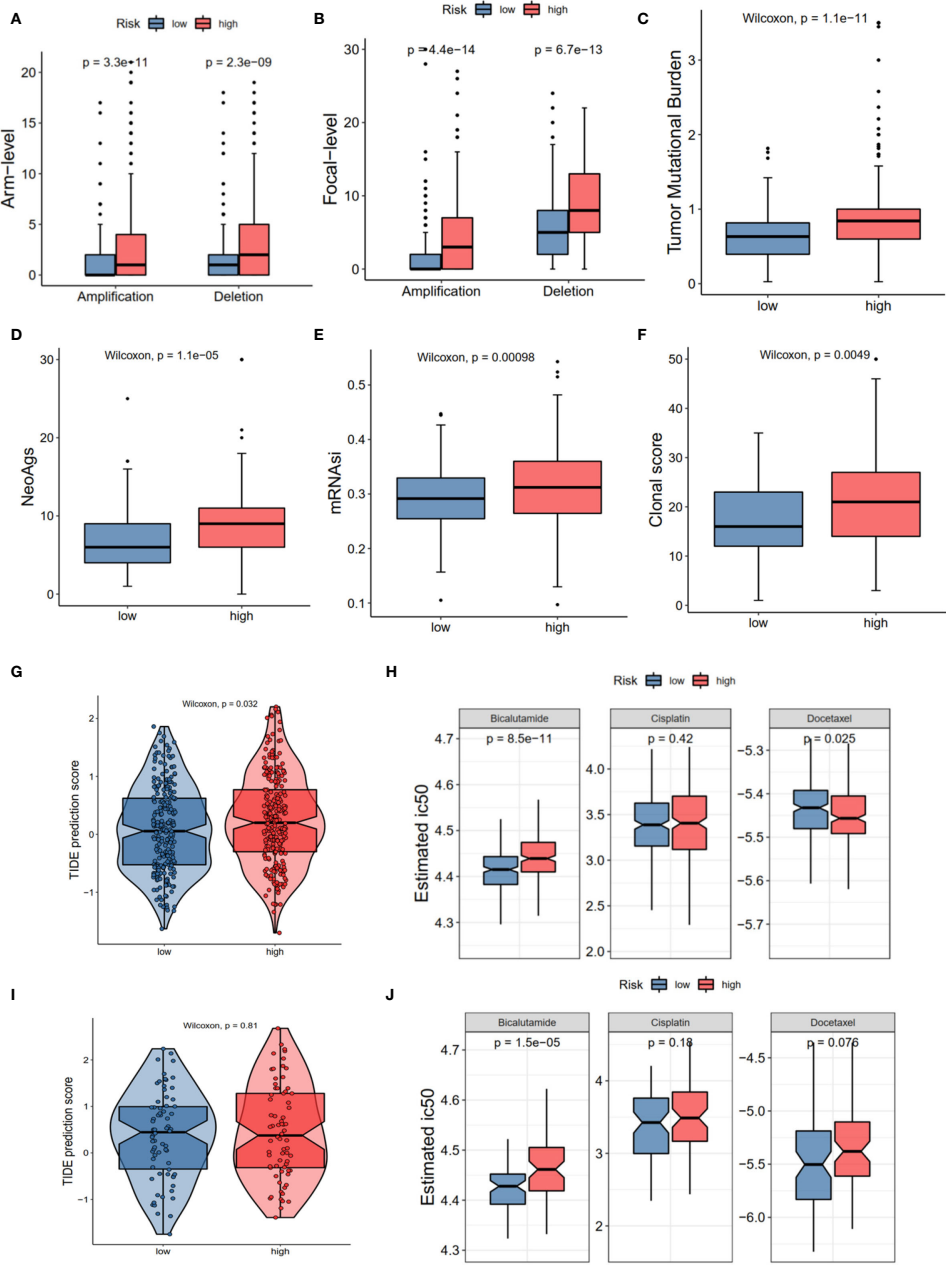
The correlations of different risk group with copy number alterations, tumor mutation burden, neo-antigens, tumor stemness, clonal status and immune-/chemotherapy response. **(A)** Arm-level copy number amplification and deletion. **(B)** Focal-level copy number amplification and deletion; **(C)** Tumor mutant burden difference. **(D)** Neo-antigens. **(E)** Tumor stemness difference represented by the mRNAsi. **(F)** Clonal status. **(G, H)** Immunotherapy response based on TIDE website and estimated IC50 indicates the efficiency of chemotherapy in TCGA. **(I, J)** Immunotherapy response and estimated IC50 in MSKCC dataset. All the survival analysis are based on the recurrence free survival data.

The GO and KEGG enrichment results showed that the risk score was closely associated with the immune process and drug metabolism pathways. The CNV, TMB, cancer stemness index and clonal score were included to determine whether the risk score was correlated with the immune response and chemotherapy. The high-risk group of the CNV status in the TCGA cohort had both high amplification (P_amp_ = 3.3e-11, P_amp_ = 4.4e-14) and deletion (P_del_ = 2.3e-09, P_del_ = 6.7e-13) in the arm and focal levels (**Figures 6A, B**). Moreover, the high-risk group in TCGA dataset had high TMB (P = 1.1e-11 **Figure 6C**), neoantigens burden (P = 1.1e-05, **Figure 6D**), mRNAsi (P < 0.001, **Figure 6E**), and clonal score (P < 0.001, **Figure 6F**). Notably, TMB and neoantigens were potential immunotherapy response biomarkers (28). Subsequently, the immunotherapy response and predictive responses of the three common chemotherapy drugs, cisplatin, docetaxel and bicalutamide were analyzed using the TIDE and GDSC database. The results revealed that the TIDE score was higher in the high-risk group (P = 0.032, TCGA dataset, **Figure 6G**), while there was no significant difference in the MSKCC dataset (P = 0.81, **Figure 6I**). Furthermore, patients in the high-risk group appeared to benefit more from anti-PD-1/PD-L1 immunotherapy than did the low-risk group (P = 0.0299, P = 0.2657, **Figure S1E**). The high-risk group had a high estimated IC50 for bicalutamide (P = 8.5e-11 TCGA; P = 1.5e-05 MSKCC) and low estimated IC50 for docetaxel in TCGA (P = 0.025), and a high estimated IC50 for docetaxel in the MSKCC (P = 0.076) (**Figures 6H, J** **)**.

### *TFRC* Overexpression Facilitates Proliferation, Migration and Invasion in PCa Cell Lines

**Figure 7:**
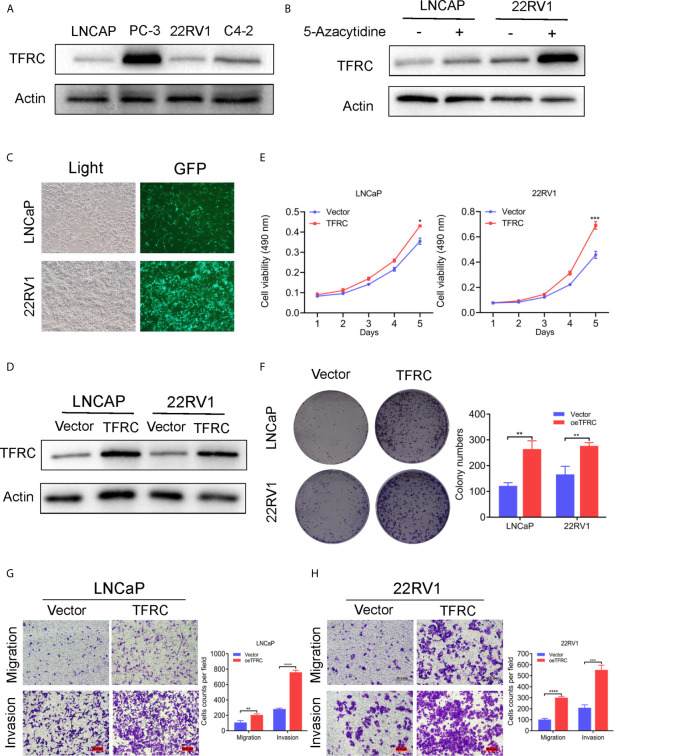
TFRC expression influenced by 5-zacytidine and overexpressing TFRC promotes proliferation, migration and invasion in Pca cells 22RV1 and LNCaP. **(A)** The protein levels of TFRC in Pca cells (C4-2, LNCaP, 22RV1 and PC-3) were detected by western blots. **(B)** 5-azacytidine inhibited DNA methylation and increased the expression of TFRC in LNCaP and 22RV1 cells. **(C, D)**. The efficiency of TFRC overexpressed lentivirus were confirmed using fluorescence microscope and western blots. **(E, F)**. The MTT and colony formation assays indicated that TFRC promotes cells proliferation in LNCaP and 22RV1. **(G, H)**. Transwell assays suggested that TFRC promotes cells migration and invasion in LNCaP and 22RV1. *P < 0.05, **P < 0.01, ***P < 0.001, ****P < 0.0001.

The hub genes could have more effect on the biological functions since the risk model was strongly associated with the PCa RFS. Therefore, we chose the high genetic alteration gene *TFRC* to confirm our hypothesis. We first measured the baseline protein levels of *TFRC* in PCa cells PC-3, LNCaP, 22RV1, and C4-2 using western blots. *TFRC* expression was relatively lower in LNCaP and 22RV1 cells compared to other cell lines (**Figure 7A**). Therefore, we used these two cell lines to overexpress *TFRC* in later experiments. Interestingly, the mRNA expression of *TFRC* decreased in PCa tissues (**Figures 1C, E** **)** compared with the normal samples, but increased in tissues with high Gleason scores and lymph nodes metastases (**Supplementary S1A, 1B**). Several investigators have reported that methylated CpG sites can block transcription initiation by inhibiting transcription factor binding, such as promoters and distal regulatory regions, then changing the mRNA expression of host genes (29–31). We explored the methylation levels of *TFRC* and found that *TFRC* had high methylation levels in PCa tissues (although the P value was not significant, **Supplementary S1C**), which were negatively associated with the mRNA expression of *TFRC* (**Supplementary S1D**). Therefore, we treated the PCa cells with 5-zacytidine [a widely-used DNA methylation inhibitor (32)] and found that the protein level of *TFRC* was increased after cells were incubated with 0.5 μM 5-zacytidine for 24 h (**Figure 7B**). We then overexpressed *TFRC* in 22RV1 and LNCaP cells (**Figures 7C, D**). The effect of *TFRC* on PCa cells was evaluated using MTT, colony formation and transwell assays. As shown in **Figures 7E, F**, upregulation of *TFRC* induced proliferation in PCa cells. Similarly, the transwell assays indicated that *TFRC* promotes the migrative and invasive activities of LNCaP and 22RV1 cell lines (**Figure 7G**).

## Discussion

Selective induction of cancer cell death is the most effective therapeutic method for tumors (33). Studies have reported that ferroptosis, a common selective induction cell death, plays pivotal role in tumorigenesis (34, 35). However, its importance in prostate cancer has not yet to be elucidated. In this study, we collected the RNA-seq data to investigate variations in mRNA expression profiles of FRGs in prostate cancer. We used univariate Cox, lasso regression, and multivariate Cox analyses to identify the signature of seven RFGs.

It has been widely reported that the seven hub genes, *AKR1C3*, *ALOXE3*, *ATP5MC3*, *CARS1*, *MT1G*, *PTGS2*, and *TFRC* are involved in the pathogenesis of several diseases. *AKR1C3*, a crucial androgenic enzyme, can reprogram AR signaling in advanced prostate cancer (36) and is involved in the production of aromatase substrates in breast cancer (37). *ALOXE3*, epidermal LOX type 3, converts fatty acid substrates to specific epoxyalcohol derivatives using R-hydroperoxides (38), is involved in late epidermal differentiation (39) and ichthyosis (40). *ATP5MC3*, also referred to as *ATP5G3* (41), encodes a mitochondrial ATP synthase subunit, and is associated with overall survival in clear cell renal carcinoma patients (42). *CARS1*, cysteinyl-TRNA synthetase 1, is associated with tRNA function and contributes to the development of inflammatory myofibroblastic tumors (43) and kidney cancer (44). *CARS1* knockdown has been proven to suppress ferroptosis induced by cysteine deprivation and promote the transsulfuration pathway (45). *MT1G*, a small-molecular weight protein that has high affinity for zinc ions, can inhibit proliferation by increasing the stability and transcriptional activity of p53 (46). *PTGS2*, also known as cyclooxygenase 2, is associated with prostanoid biosynthesis (47). Several studies have demonstrated that *PTGS2* induces cancer stem cell-like activity and promotes the proliferation, angiogenesis and metastasis of cancer cells (48–50). *TFRC* is a cell surface receptor necessary for cellular iron (51). Few of these genes have been investigated in PCa (except PTGS2 and MT1G). In this study, all seven genes were shown to correlate with RFS in patients with PCa.

The ferroptosis-related risk model was constructed in the TCGA PCa cohort using the mRNA expression profiles of these seven hub genes. The KM plot and ROC curve showed that this risk score could easily predict long versus short RFS. These findings were also validated in the MSKCC external validation dataset. The PCa patients were divided into two groups according to the risk score to further explore the detailed FRG mechanisms. There were 664 DEGs in these two groups. Interestingly, these DEGs were enriched in immune-related GO terms and metabolism KEGG terms (**Figures 5B, C**). Subsequently, the immune infiltration status of 28 immune cells was calculated using the ssGSEA method, and the results showed that several immune cells, such as central memory CD8 T cell, CD56^bright^ and natural killer cell were associated with the risk score (**Figures 5D–G**), suggesting that this risk model is appropriate for immunotherapy. The immunotherapy-related signatures, such as CNV load, TMB, mRNAsi, clonal score, and TIDE score were included for further investigation. These results indicated that the high-risk group was correlated with the CNV load, TMB, neoantigens, mRNAsi, and clonal score (**Figures 6A–F**), implying that the high-risk score group has a better clinical response to immunotherapy. The TIDE score in the high-risk group was also increased, indicating better outcomes for immune therapy (**Figures 6G, I** **)**. Since most KEGG terms between the low and high-risk groups were enriched in metabolism (especially in drug metabolism) (**Figure 5C**), we determined the estimated IC50 for three commonly used drugs. The results showed that there was an increase of bicalutamide in the high-risk group (**Figures 6H–J** **)**, implying that this group could be bicalutamide resistant. In other words, FRGs could have effects on RFS *via* regulating drug responses. And the high-risk patients can improve their situations through different immunotherapies, providing a potential strategy for the individual treatment of PCa patients.

Finally, we chose *TFRC*, which had a high genetic alteration rate, for further validation. Since the mRNA level of *TFRC* was lower in PCa patients compared with normal tissues, we overexpressed *TFRC* to determine its effects in PCa cells. LNCaP and 22Rv1 cell lines were selected based on the baseline protein expression levels (**Figures 7A, C, D**). MTT, clonal formation and invasion assays showed that *TFRC* overexpression can significantly increase the proliferation (**Figure 7F**) and invasion (**Figure 7G**) of PCa cells. We also added 5-Aza to inhibit DNA methylation of *TFRC*, which demonstrated that the hypermethylation status of the *TFRC* gene induces a relatively low PRAD tissue expressions, but increases the trend of the Gleason score. This finding suggests that *TFRC* can play an oncogenic role in PCa, but the detailed mechanism needs to be further explored.

In summary, our study systematically evaluated the expression of FRGs and their potential prognostic value in PCa. Moreover, a risk model of seven FRGs was established in TCGA and validated using the MSKCC dataset. We found that the high-risk group was correlated with elevated CNV load, TMB, neoantigens, mRNAsi, clonal score, and immunotherapy response, but also with a high estimated IC50 for bicalutamide. Finally, we assessed the effects of overexpressed *TFRC* on proliferation and invasion in PCa cell lines.

This study has several limitations. First, our risk model was established and validated based on public databases, more prospective real-world data are needed to confirm its clinical significance. Second, we only initially explored the effect of *TFRC* overexpression on biological functions, the specific mechanism *in vivo* and *in vitro* should be investigated for further.

## Conclusions

We established a ferroptosis-related risk model, that was strongly associated with aberrant CNV load, TMB, mRNAsi, neoantigens, and clonal score. In addition, we validated TFRC to be an oncogenic factor in prostate cell lines. Our research provides new insights into personalized therapies for PCa patients.

## Data Availability Statement

The datasets presented in this study can be found in online repositories. The names of the repository/repositories and accession number(s) can be found in the article/**Supplementary Material**.

## Author Contributions

YX and LG: conceptualization, supervision, methodology, and writing -review & editing. HL and LG: data curation, formal analysis, and writing-original draft. JL, T-sZ, and TX: validation, visualization, and investigation. All authors contributed to the article and approved the submitted version.

## Funding

This work was supported by the National Natural Science Foundation of China (grant NO.81971371).

## Conflict of Interest

The authors declare that the research was conducted in the absence of any commercial or financial relationships that could be construed as a potential conflict of interest.

## Supplementary Material

Supplementary Figure 1 The associations between TFRC mRNA expression levels and Gleason score **(A)**, nodal metastasis **(B)** and methylation levels **(C, D)** in TCGA PCa patients. Subclass mapping (SubMap) analysis of patients respond to anti–PD-1/PD-L1 treatment **(E)**. Multiple ROC (Receiver Operating Characteristic) in TCGA and MSKCC **(F)**. All the survival analysis are based on the recurrence free survival data.
