# Supplementary figures and images for: Identification and Validation of a Prognostic Signature for Prostate Cancer Based on Ferroptosis-Related Genes
Source: Front Oncol. 2021 Jul 15;11:623313. doi: 10.3389/fonc.2021.623313 (PMC8320699; doi:10.3389/fonc.2021.623313)

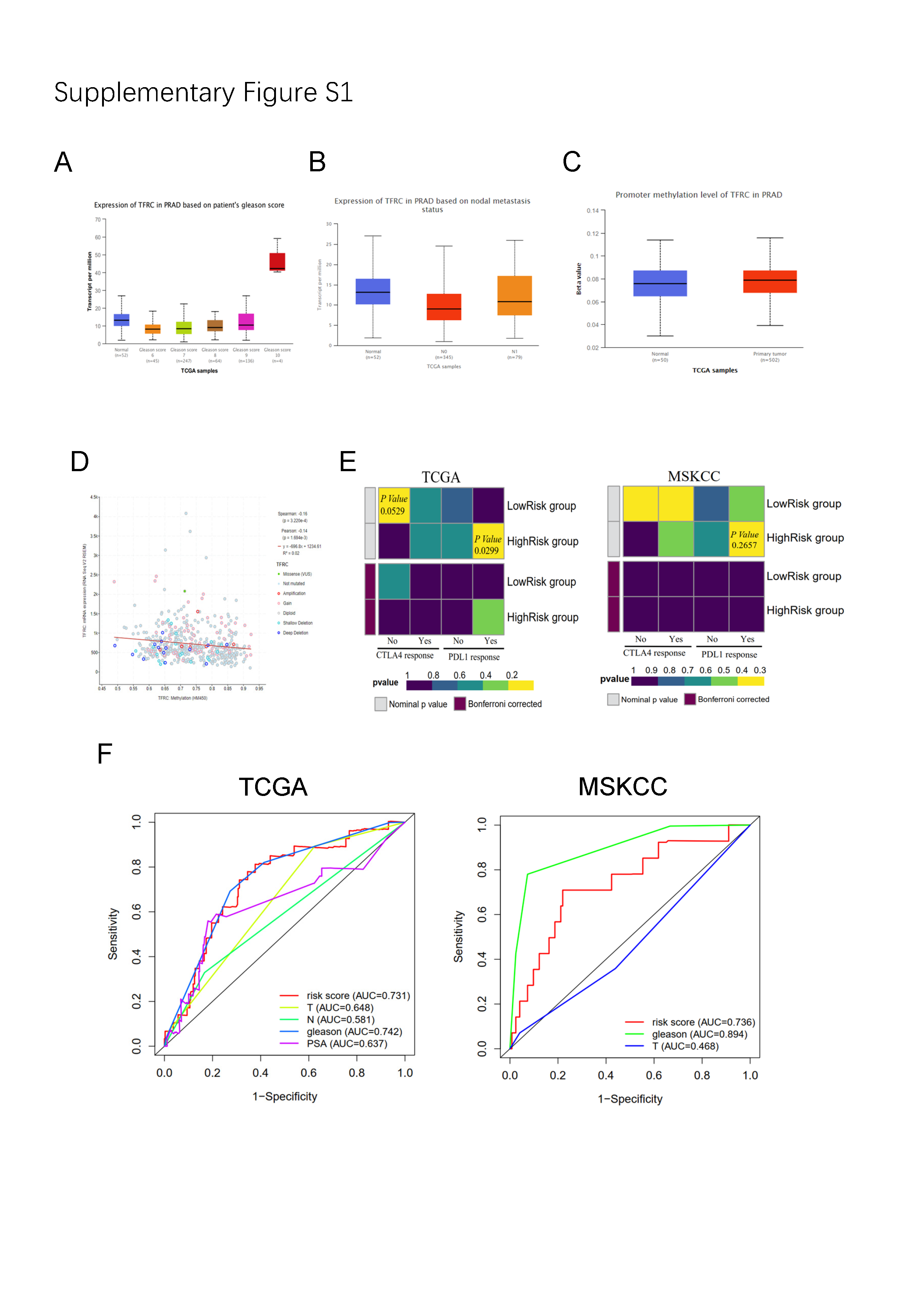

Supplement: Supplementary Figure 1 — The associations between TFRC mRNA expression levels and Gleason score (A), nodal metastasis (B) and methylation levels (C, D) in TCGA PCa patients. Subclass mapping (SubMap) analysis of patients respond to anti–PD-1/PD-L1 treatment (E). Multiple ROC (Receiver Operating Characteristic) in TCGA and MSKCC (F). All the survival analysis are based on the recurrence free survival data. [file Image_1.tiff]
